# Duodenoscope combined with laparoscopy in treatment of biliary stones for a patient with situs inversus totalis: A case report

**DOI:** 10.1097/MD.0000000000014272

**Published:** 2019-02-15

**Authors:** Liangshuo Hu, Yichao Chai, Xue Yang, Zheng Wu, Hao Sun, Zheng Wang

**Affiliations:** Department of Hepatobiliary Surgery, First Affiliated Hospital of Xi’an Jiaotong University, Xi’an, Shaanxi, China.

**Keywords:** choledocholithiasis, cholelithiasis, endoscopic retrograde cholangiopancreatography, laparoscopic cholecystectomy, situs inversus totalis

## Abstract

**Rationale:**

Although endoscopic and laparoscopic techniques in situs inversus totalis (SIT) have been reported respectively, endo-laparoscopic combination therapy due to biliary lithiasis remains infrequent. We shared the experience regarding the operations with a video report and discussed the similarities and differences with the usual procedures, which proved to be challenging to some extent for SIT.

**Patient concerns:**

Herein we present a 72-year-old man with SIT who underwent endo-laparscopic combination therapy due to choledocholithiasis and gallbladder stone.

**Diagnosis:**

Choledocholithiasis; Gallbladder stone; SIT

**Interventions:**

The patient underwent endoscopic retrograde cholangiopancreatography (ERCP) first. He was placed in the left lateral decubitus position with basal anesthesia. As a result of the anatomical abnormality, the endoscope was rotated 180° in the 2nd portion of the duodenum. The ampulla was identified with difficulty because of a giant duodenal diverticulum nearby. After double-wire-guided cannulation, cholangiogram demonstrated filling defects and sphincterotomy was performed. This was followed by balloon ampulla dilation, sludge sweepage and nasobiliary drainage. The patient underwent standard laparoscopic cholecystectomy (LC) the next day.

**Outcomes:**

No complications such as bleeding, pancreatitis, perforation (after ERCP) or bile leakage (after LC) was detected. The patient was discharged after 4 days and recovered well after 3 months follow-up.

**Lessons:**

We found that patients were not required to make changes in position; the medical staff should adapt to mirror symmetrical anatomy and operate carefully. The surgical outcomes were not affected despite the extended operation time. In addition, operators can amend usual operating habits with modified techniques for patients with SIT.

## Abstract 2

Supplemental Digital Content is available in the text

## 1 Introduction

Situs inversus is a generally autosomal congenital recessive genetic condition that results from abnormalities of deletions on chromosomes 7 and 8,^[1]^ and sometimes attributed to identical mirror image twins, which is characterized by mirrored visceral organs from their normal positions. Epidemiologically, the morbidity rate presents no fewer than 1/20,000 live births in the general population, with a male/female ratio of 3:2.^[2]^ At present, there are over 1360 retrieval published medical reports in the PubMed databases. Currently, it is acknowledged that situs inversus sometimes may be associated with other congenital anomalies, such as renal dysplasia, biliary atresia, congenital heart disease or pancreatic fibrosis,^[3]^ but most people with situs inversus do not also have medical symptoms. However, their special visceral locations require particularly cautious diagnosis and therapy by medical staff, especially surgical treatment. Here we reported a case of situs inversus totalis (SIT) who underwent endo-laparscopic combination therapy for choledocholithiasis and a gallbladder stone to share our therapeutic experience.

## 2 Case presentation

**Figure 1:**
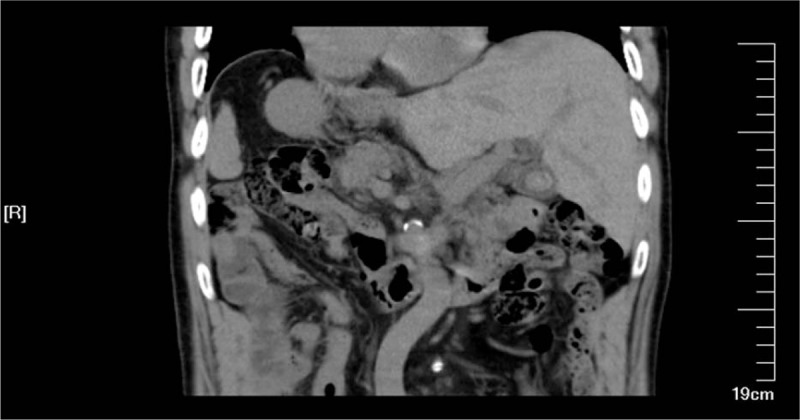
Computed tomography (CT) scan confirmed the diagnosis of situs inversus totalis (SIT) with gallbladder stone.

**Figure 2:**
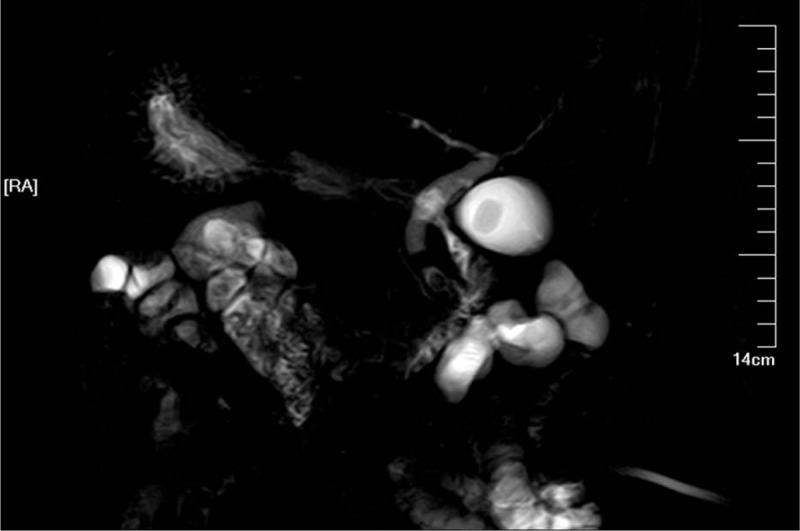
Magnetic resonance cholangiopancreatography (MRCP) scan confirmed the diagnosis of choledocholithiasis and gallbladder stones.

A 72-year-old man was admitted to the local hospital due to repeated pain in epigastric region three months ago. He was diagnosed as acute pancreatitis with SIT and underwent conservative treatment. After this period, he visited our hospital due to recurrent pancreatitis. While treating acute pancreatitis, computed tomography (CT) and magnetic resonance cholangiopancreatography (MRCP) scan confirmed the diagnosis of SIT with choledocholithiasis and gallbladder stone (Figs. 1 and 2). After comprehensively analyzing the cause, we noted that pancreatitis may relapse again in the future if cholelithiasis is not eradicated. In consideration of the patient's age, he underwent laparoscopic cholecystectomy (LC) combined with endoscopic choledocholithotomy after his symptoms were relieved.

**Figure 3:**
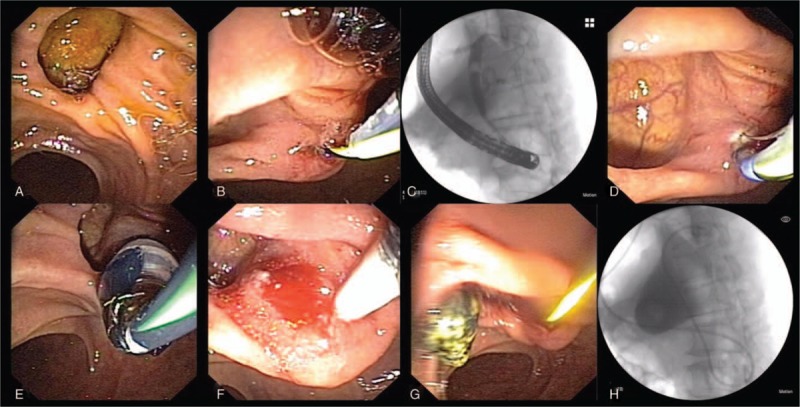
Therapeutic endoscopic retrograde cholangiopancreatography (ERCP) was performed for choledocholithiasis. (A) Giant duodenal diverticulum; (B) exhaust air for cholangiography; (C) filling defects; (D) sphincterotomy; (E) endoscopic papillary balloon dilation (EPBD); (F) inserting balloon catheter; (G) dilation assisted stone extraction (DASE); (H) endoscopic naso-biliary drainage (ENBD).

The patient underwent endoscopic retrograde cholangiopancreatography (ERCP) first. He was placed in the left lateral decubitus position with basal anesthesia (An additional video file shows this in more detail [Additional file 1]). As a result of the anatomical abnormality, the endoscope was rotated 180° clockwise crossing the stomach and introduced to the second portion of duodenum. The ampulla was identified with difficulty because of a giant duodenal diverticulum nearby (Fig. 3**A**). The endoscope was rotated clockwise 30° without torsion under fluoroscopic guidance. After double-wire-guided cannulation and exhaust air for cholangiography (Fig. 3**B**), the cholangiogram showed filling defects (Fig. 3**C**) and sphincterotomy was performed (Fig. 3**D**). This was followed by endoscopic papillary balloon dilation (EPBD) (Fig. 3**E**), inserting a balloon catheter for sweep of sludge (Fig. 3**F**) and dilation assisted stone extraction (DASE) procedure (Fig. 3**G**). An endoscopic naso-biliary drainage (ENBD) was placed (Fig. 3**H**).

**Figure 4:**
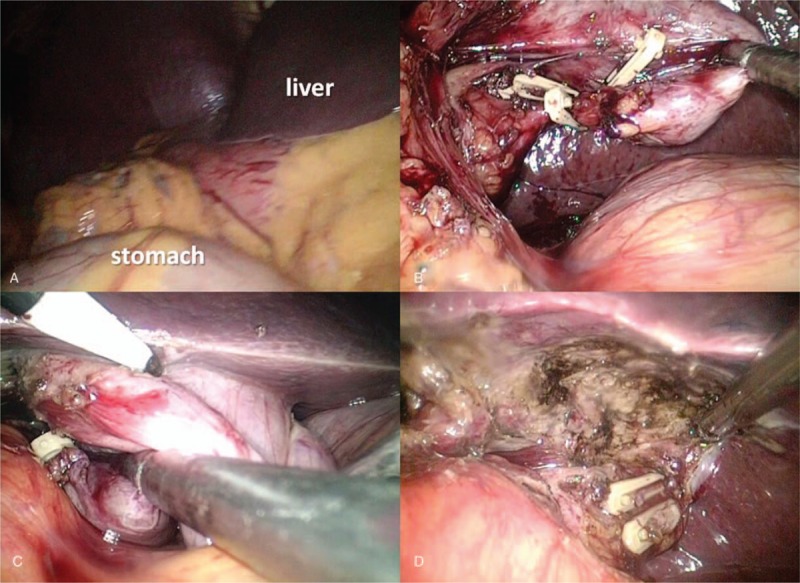
Laparoscopic cholecystectomy (LC) with situs inversus totalis (SIT). (A) Mirrored visceral organs from their normal positions; (B) cystic artery and duct were clipped by clips; (C) the gallbladder was excised in retrograde fashion; (D) hemostasis of gallbladder bed.

On the second day, the patient had no discomfort and underwent standard LC (An additional movie file shows this in more detail [see Additional file 2]). He was in supine position, with his head up and feet down at an angle of 30° and leaning 15° left. The surgeon and the mirror-gripping assistant were on the left side of the patient, and the 1st assistant was on the right side. The classical 4-ports technique was used, but at sites opposite the usual ones. There was 2 10 mm Trocar of ports (periumbilical) and 2 5 mm Trocar ports (midclavicular and anterior axillary line on the left epigastric). On the intraabdominal inspection, we could observe that the liver and the gallbladder were located on the left side of the patient, and the stomach was on the right side (Fig. 4**A**). Calot's triangle was dissected above the plane of Rouviere's sulcus, which was located at the left side of porta hepatis. A grasper was used to pull the ampulla of gallbladder to left to expose Rouviere's sulcus and to reach the common bile duct position. The cystic artery and duct were clipped by absorbable clips and titanium clips (Fig. 4**B**) and cut off to enhance the operative speed. Finally, the gallbladder was excised in retrograde fashion (Fig. 4**C**) and then, after the gallbladder bed reached hemostasis, extracted through the subumbilical port (Fig. 4**D**). The drainage tube was placed at the gallbladder fossa and the abdominal wall was closed as usual. The total operation time was 40 min and blood loss was 20 ml. The patient recovered well and the drainage tube and ENBD tube were removed on the 2nd postoperative day. No complications such as bleeding, pancreatitis, perforation (after ERCP) or bile leakage (after LC) was detected. He was discharged 4 days after the operation and had recovered well as of 3 months follow-up.

## 3 Discussion

Therapeutic endoscopic retrograde cholangiopancreatography (ERCP) and laparoscopic cholecystectomy (LC) are standard procedures for biliary stones. The same principles apply for the patients with situs inversus, which is not a risk factor for cholelithiasis by itself, but carries a risk of surgery, because the reversed organs give rise to reversal of the surgical habits and techniques of surgeons, and increase surgical difficulty.^[3]^

To overcome the technical difficulty caused by anatomical right-left reversal and increase the success rate, several techniques have been introduced for ERCP in patients with situs inversus. Some studies reported the technique with endoscope rotated 180° clockwise in the stomach. However, this technique requires skilled endoscopist and may be difficult in cannulating when anatomic variation exist at the ampulla (e.g. periampullary diverticulum, Billroth II anatomy).^[4,5]^ Reversal of the position for the endoscopist in relation to the patient is another way to overcome the anatomical difficulty,^[6]^ but there also have been some successful cases of ERCP that suggest that modified patient position is not necessary. This is consistent with our experience.^[7]^ Duodenal diverticula is present in up to 25% of patients and rarely cause symptoms, but it may become a challenge in ERCP surgery, especially in patients with SIT due to altered anatomy.^[8]^ We found that papilla identification and cannulation are difficult in SIT patients with periampullary diverticulum.^[9]^ The double-wire-guided technique may increase the success rate of cannulation.^[10]^

One technical challenge of LC for patients with SIT is the location of the ports. It is not convenient for a right-handed surgeon to perform cholecystectomy only with a 5 mm Trocar placed below the left costal margin, 4-ports technique was used for more flexible coordination of the traction gallbladder. Similarly, 3-ports technology could also be suitable.^[11]^ Another difficulty is that the associated anomalies with SIT of the coeliac trunk andliver are reported to be very common in individuals with situs inversus.^[12]^ Vascular anomalies may cause unwilling hemorrhage and confusion and make the operation more challenging. Surgeons should also adapt to operate in the opposite direction using pre-simulated exercise to prevent iatrogenic bile duct injuries. Cholangiogram is recommended to confirm the inverse anatomy and exclude further anatomical variants.^[13]^

In summary, we showed the management of choledocholithiasis with gallbladder stone by endo-laparscopic combined surgery in a patients with SIT. Limitation of this study is that most of the techniques we discussed are based on single center experience. Previous reports are mostly case reports and a prospective study is still required to determine the optimal approach for patients with SIT.

## 4 Conclusion

Endo-laparscopic combination in treating choledocholithiasis with gallbladder stone was applicable to patients with SIT, and this did not require position changes. Surgeons need to carefully manipulate according to the new anatomical adjacent relationships and structures. They can also adapt their usual operating habits with modified techniques for patients with SIT.

## Author contributions

LH and YC recorded the view data as the joint 1st authors who had wrote this paper; XY and HS performed an operative treatment of choledocholithiasis by an ERCP; Z Wu and Z Wang completed the surgical procedure of laparoscopic cholecystectomy, Z Wang served as the patient's attending physician; all authors participated in the revision of the manuscript and read and approved the final manuscript.

**Conceptualization:** Zheng Wang.

**Formal analysis:** Liangshuo Hu, Yichao Chai, Zheng Wang.

**Investigation:** Zheng Wang.

**Methodology:** Xue Yang, Zheng Wu, Hao Sun.

**Project administration:** Zheng Wang.

**Resources:** Xue Yang, Zheng Wu, Hao Sun, Zheng Wang.

**Supervision:** Xue Yang, Zheng Wu, Hao Sun.

**Visualization:** Liangshuo Hu, Yichao Chai.

**Writing – original draft:** Liangshuo Hu, Yichao Chai.

**Writing – review & editing:** Zheng Wang.

## Supplementary Material

Supplemental Digital Content

## Supplementary Material

Supplemental Digital Content
